# Influence of water evaporation/absorption on the stability of glycerol–water marbles†

**DOI:** 10.1039/c9ra05728e

**Published:** 2019-10-28

**Authors:** Xinxing Lin, Wei Ma, Lihui Chen, Liulian Huang, Hui Wu, Atsushi Takahara

**Affiliations:** Fujian Agriculture and Forestry University Fuzhou Fujian 350002 China wuhui@fafu.edu.cn fafuclh@163.com +86-591-83715175 +86-18649784585; International Institute for Carbon-Neutral Energy Research (WPI-I^2^CNER), Kyushu University 744 Motooka Nishi-ku Fukuoka 819-0395 Japan; ERATO Takahara Soft Interface Project, Japan Science and Technology Agency (JST) 744 Motooka Nishi-ku Fukuoka 819-0395 Japan

## Abstract

The porous shell structure of liquid marbles allows liquid vapor to enter in/out of the liquid marbles, leading to the deformation/collapse of liquid marbles, which limits their application as miniature reactors for long-term chemical reactions. In this study, to prevent volatilization and maintain long-term stability, stable liquid marbles were fabricated by encapsulating glycerol/water droplets using superhydrophobic cellulose nanocrystals. The influence of water evaporation and absorption on the stability of aqueous glycerol marbles at different relative humidities (RHs) was investigated. At the same RH, the evaporation/absorption rates of the liquid marbles decreased on increasing the glycerol concentration. For the liquid marbles with the same glycerol volume concentration, the evaporation rates decreased with the increase in RH. The liquid marbles exhibited higher evaporation/absorption resistance compared with pure naked liquid droplets.

## Abstract 2

The influence of water evaporation and absorption on the stability of aqueous glycerol marbles was investigated.

## Graphical abstract

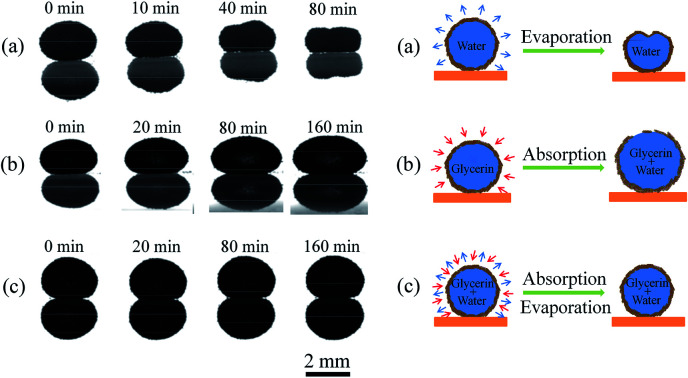

## Introduction

Liquid marbles are droplets encapsulated with a protective armor of micro- or nano-scaled particles at the liquid–gas interface.^1^ Liquid marbles have attracted significant attention because of their unique morphology and properties.^2–8^ Because the armored particles prevent a direct contact between the liquid and the substrate, the marbles can not only roll off the solid substrate without any leakage, but also float on the liquid surface.^9–14^ Since the droplets are in the non-sticky state, liquid marbles can be manipulated easily by gravitational, electrostatic, and magnetic fields and light depending on the components.^15–21^ The marble can bounce off a solid surface when it falls from a certain height, exhibiting high stability on impact. Under the action of external forces, a liquid marble can be split into two smaller liquid marbles without the spreading of liquids, showing a unique self-healing behavior.^19,22^ When two liquid marbles are placed in contact, coalescence may occur by different coalescence techniques such as impact, magnetic force, vertical collision and direct-current voltage.^23–27^ Given the flexibility in the choice of solid particles and liquids to fabricate liquid marbles, liquid marbles are emerging as a versatile platform for a wide range of applications in miniature reactors, microfluidics, drug transport, gas sensing, and biomedical and genetic analyses.^2–6^

As a versatile and cost-effective miniature reactor providing a well-confined microenvironment,^28–32^ progress in miniaturized chemical and biological processes using liquid marbles has been reported recently. The miniaturized synthesis of graphene/Ag nanocomposites was demonstrated using liquid marbles.^28^ Silica-stabilized liquid marbles were employed as a substrate to carry out the interfacial silver mirror reaction to prepare Janus particles.^29^ Carbon dioxide (CO_2_) gas was controlled to initiate multiple micro-reactions in parallel *via* the coalescence of contacting liquid marbles.^30^ However, the porous shell structure of the liquid marbles allows the liquid vapor to enter in/out of the liquid marbles, leading to the deformation/collapse of liquid marbles, which limit their application in miniature reactors for long-term chemical reactions. Thus, it is a big challenge to prevent volatilization and maintain long-term stability for the long service life of liquid marbles.

The evaporation or absorption of liquids is crucial to control the morphology of liquid marbles. The layer organization of coatings and types of liquids can significantly influence the evaporation/absorption of liquids.^33–41^ When the liquid marble is covered with a monolayer coating of polystyrene particles, the evaporation rate is higher than that of a naked water droplet.^33^ The liquid marble with a multilayered coating has a lower evaporation rate than bare liquid droplets due to the presence of evaporation resistance.^34,35^ Besides the coating particles, the volatility of the encapsulated liquid is also an important factor for the stability of liquid marbles. If the coated liquid, such as glycerol and ionic liquids, possesses excellent hygroscopicity, we can anticipate that the volume of the marble will increase owing to the absorption of moisture. Therefore, when the evaporation rate and the absorption rate of a mixture solution are balanced, the marble can maintain a stable state. This motivated us to investigate liquid marbles with long-term stability prepared using hygroscopic liquids for the potential application of miniature reactors in miniaturized chemical processes.

Here, we proposed a strategy to fabricate liquid marbles with long-term stability by encapsulating glycerol/water droplets using superhydrophobic cellulose nanocrystal particles. The effects of water evaporation and absorption on the stability of aqueous glycerol marbles at different relative humidities (RHs) were investigated.

## Experiments

### Materials

The rod-like cellulose nanocrystals (CNC) with an average length of 263 ± 89 nm and a diameter of 24 ± 6 nm were obtained from Tianjing Woodelf biotechnology Co., Ltd, China. Ferric chloride (FeCl_3_), ferrous chloride (FeCl_2_), ammonium hydroxide (NH_4_OH), and glycerol were of analytical grade and were purchased from Shanghai Aladdin Biochemical Co., Ltd, China. Magnesium chloride (MgCl_2_), ammonium nitrate (NH_4_NO_3_), sodium nitrate (NaNO_3_), and potassium chloride (KCl) were obtained from Sinopharm Chemical Reagent Co., Ltd, China.

### Preparation of liquid marbles

The liquids with different volume concentrations of glycerol from 0 to 100% were used to prepare liquid marbles. After dropping 10 μL of liquid droplets onto a bed of poly(DOPAm-*co*-PFOEA)/Fe_3_O_4_/CNC (PFC) nanoparticles, the liquid marble was fabricated when the liquid rolls over the PFC powder. The fabrication of PFC nanoparticles was performed following the previously reported method.^19^ Briefly, the spherical Fe_3_O_4_ nanoparticles with a diameter of 8 ± 2 nm were deposited on the surface of CNC using a mixture solution of FeCl_3_ and FeCl_2_. Then, the Fe_3_O_4_/CNC nanoparticles were modified by poly(DOPAm-*co*-PFOEA) to obtain the superhydrophobic PFC nanoparticles. The weight ratio of CNC : Fe_3_O_4_ : poly(DOPAm-*co*-PFOEA) was 79.0 : 19.7 : 1.3. The PFC nanoparticles have excellent superhydrophobicity with advancing/receding water contact angles of 168°/166°.

### Characterization

The gravimetric measurements of liquid marbles with different glycerol concentrations at various RHs were conducted using an electric balance (AUW120D). The electric balance was placed in an air-conditioned laboratory at 297.2 ± 1.0 K under RH of 50 ± 2%. The geometry of liquid marbles was acquired using a camera (Nikon V3). The liquid marble was placed on the weighing pan of a sealed electronic balance. Four bottles of the same saturated solution were placed within the corners of the electronic balance to adjust humidity (Fig. S1 in the ESI†). The saturated solutions of MgCl_2_, NH_4_NO_3_, NaNO_3_, and KCl were used as hygrostat solutions to control the humidity of the electronic balance to 40 ± 2% RH, 60 ± 2% RH, 76 ± 2% RH, and 82 ± 2% RH, respectively.^36^ The silica gel was used to adjust the RH to 26 ± 2%. The humidity was monitored using a hygrometer. After the humidity of the electronic balance reached equilibrium, the liquid marble with a volume of 10 μL was quickly transferred into the electronic balance. The mass of the liquid marble was recorded at an interval of 4 min. The horizontal profiles of liquid marbles in a sealed home-made glass box were determined using a DSA30 contact angle measurement instrument. The surface morphology of the liquid marble was observed using an optical microscope (XPL-60) in the reflection mode. The area percentage of interparticle gaps in the photographs of liquid marbles was evaluated using ImageMagick.

## Results and discussion

**Fig. 1:**
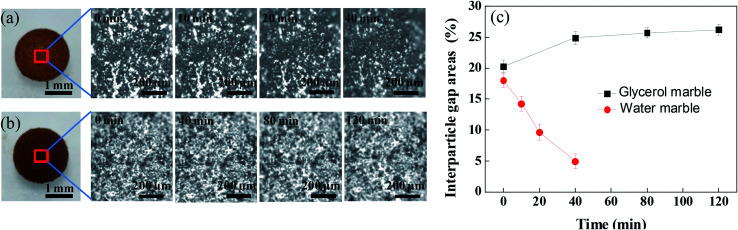
Top view of the morphology change of (a) the water marble and (b) the glycerol marble at various time intervals by optical microscopy. (c) Area percentage change of interparticle gaps of glycerol marble and water marble at different time intervals.

The coating structure of liquid marbles has a significant influence on the evaporation of the coated liquid droplet. The thickness of the coating estimated by the gravimetric contrast before and after the formation of the liquid marble was 20 ± 6 μm, which was much larger than the size of the PFC nanoparticles. Therefore, PFC nanoparticles on the surface of the water droplet formed a multilayered shell. The aggregation morphology of the PFC nanoparticles in the surface of the liquid marble was investigated using an optical microscope. As observed under the reflection mode, the aggregated PFC nanoparticles in the coating of the liquid marble appear as dark dots, while the white areas between the dark dots are interparticle gaps, as shown in Fig. 1. The appearance of white interparticle gaps clearly showed that the surface of the liquid marble was not well-covered by the PFC particles, indicating that a porous shell was present on the surface of the liquid droplet. Due to the attractive cohesion forces (van der Waals forces)^42,43^ between the PFC particles, the PFC particles distributed loosely on the surface of the liquid marble rather than in a dense film state in the liquid–gas interface. This allowed gas exchange, evaporation and absorption to take place for the coated liquid droplet.

The volume of the liquid marble decreases with time if the encapsulated liquid, such as water and ethanol, is volatile. For the water marble (Fig. 1a), the area of the interparticle gaps shrinked with time. Through the movement between hydrophobic particles, the area percentage of interparticle gaps decreased from 18.6% to 4.9% within 40 min, showing that the particles aggregated severely. In contrast, when the encapsulated liquid was hygroscopic, the volume of the liquid marble gradually increased with time. Especially, glycerol is a hydrophilic liquid with strong water-absorbing capacity. As expected, the wrapped glycerol absorbed the moisture from the environment, resulting in increase in volume. The area percentage of the interparticle gaps increased from 20.3% to 26.2% (Fig. 1b).

**Fig. 2:**
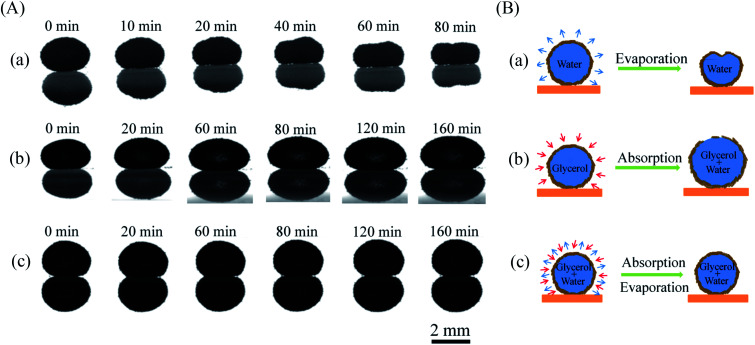
(A) Horizontal profiles and (B) schematic of (a) pure water marble, (b) pure glycerol marble, and (c) glycerol–water marble during evaporation in 50% RH.

To study the shape evolution of a liquid marble during evaporation, we used a drop shape analyzer to observe the morphology of different liquid marbles encapsulated with water, glycerol, and a water/glycerol mixture. Fig. 2a shows the horizontal profile evolution of water marbles with time during evaporation. The water marble was approximately in the shape of a sphere initially. The volume of the liquid marble gradually diminished with water evaporation. The height reduced evidently, and the shape became irregular and finally collapsed. On the contrary, the volume of the liquid marble gradually increased when the encapsulated liquid was glycerol (Fig. 2b). However, for the water–glycerol mixture marble with a certain glycerol content (Fig. 2c), the marble could maintain its original shape for a long time. An equilibrium state of the absorption and evaporation rates of water was achieved, and the mass of the liquid marble did not change.

**Fig. 3:**
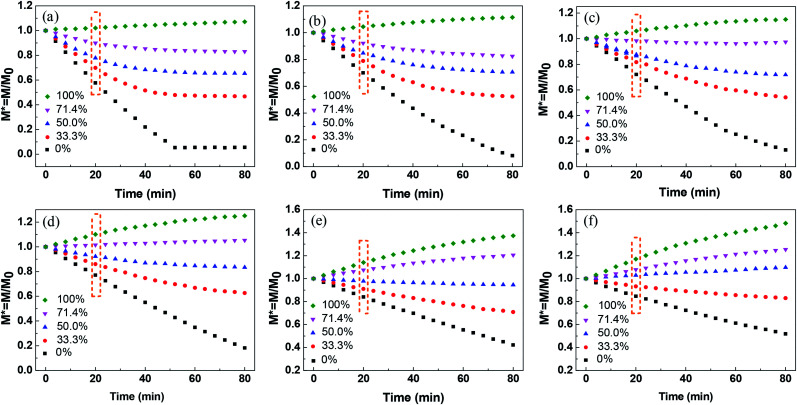
Normalized mass of liquid marbles at different glycerol concentrations under relative humidity of (a) 26%, (b) 40%, (c) 50%, (d) 60%, (e) 76%, and (f) 82%.

The liquid evaporation/absorption rate is crucial to control the stability of liquid marbles. To study the influence of water evaporation/absorption on the morphology of the liquid marble quantitatively, the mass of liquid marbles with different volume concentrations of glycerol at various RHs was measured using an electric balance. The morphological evolution of the liquid marble was demonstrated by the change in the normalized mass, as shown in Fig. 3a–f. The normalized mass is given as^39^

1 M* = *M*/*M*_0_

where *M* is the instantaneous mass and *M*_0_ is the initial mass.

Fig. 3a shows the normalized mass of the liquid marble with different glycerol volume concentrations at 26% RH. The slope of the curves (dM*/d*t*) corresponds to the change in M* with time, showing the difference in the evaporation rate and the absorption rate of water from the liquid marble. If the slope is negative, the evaporation rate is higher than the absorption rate of water, showing that the evaporation rate dominates the morphological change in the liquid marble. In contrast, a positive slope indicates that the absorption governs the weight changes. For the pure water marble with a glycerol content of 0%, the normalized mass of the liquid marble decreased within 52 min, indicating that the water marble lost weight, and water in the liquid marble evaporated continuously. However, M* levelled off with time after 52 min, indicating that almost no water was left in the marble and the liquid marble collapsed.

When glycerol was added into the liquid marble and the volume concentration of glycerol was 33.3%, the curve slope was higher than that of the pure water marble. Owing to the addition of hygroscopic solutes, the solvent in the liquid phase was stabilized greatly. As water gradually evaporated from the liquid marble, the concentration of glycerol in the liquid marble increased. This led to a slower evaporation rate at the later stage and eventually reached an equilibrium state of evaporation and absorption rates at about 48 min. When the volume concentrations of glycerol increased from 33.3% to 71.4%, the slope of the curves in the initial stage increased. This shows that the liquid marbles with higher glycerol concentrations have lower water evaporation rates. Although the liquid marble with higher glycerol concentrations could absorb more moisture, the evaporation rate of water was still higher than the absorption rate at low RH, leading to a negative slope. For the pure glycerol marble with a glycerol content of 100%, the slope was found to be positive, showing that the mass of the liquid marble increased and the glycerol marble absorbed water from the environment. The mass of the marble gradually levelled off. This demonstrated that the rates of water absorption decreased as more water entered into the marble and the glycerol concentration decreased. For all the marbles, the slope was approximated linearly within the initial 24 min.

The influence of RH on the morphology of the liquid marble was also investigated. As shown in Fig. 3a–f, when RH increases from 26% to 82%, the M* slope of the pure water marble increases, indicating that the water evaporation rates decrease. Therefore, higher RH leads to slower evaporation of water and better evaporation resistance.^35,36^ This trend can be observed for liquid marbles with glycerol concentrations from 33.3% to 100%.

Long-standing stable liquid marbles are beneficial for time-consuming chemical reactions. Either collapse or inflation is not favorable to maintain a stable liquid marble. In order to maintain the long-term stability of aqueous glycerol marbles, the mass of the liquid marbles should be constant, namely, the evaporation rate should be equal to the absorption rate of the liquid. Therefore, understanding the relationship between relative humidity and glycerol concentrations is significant for controlling the morphology of liquid marbles. An empirical model is simple and valid for the prediction of the relationship between relative humidity and glycerol volume concentrations. However, the correlation of relative humidity with glycerol volume concentrations cannot be directly established since another variant, namely, time is not involved. Thus, to eliminate this factor, the relationship of normalized mass with glycerol volume concentrations for a stable liquid marble is built by employing the same evaporation time. From Fig. 3, it can be inferred that the evaporation/absorption rate of liquid marbles remains almost constant before 24 min. Therefore, the normalized mass of the liquid marble *vs.* glycerol concentrations at a fixed time before 24 min can be used to evaluate the formation of liquid marbles with long-term stability.

**Fig. 4:**
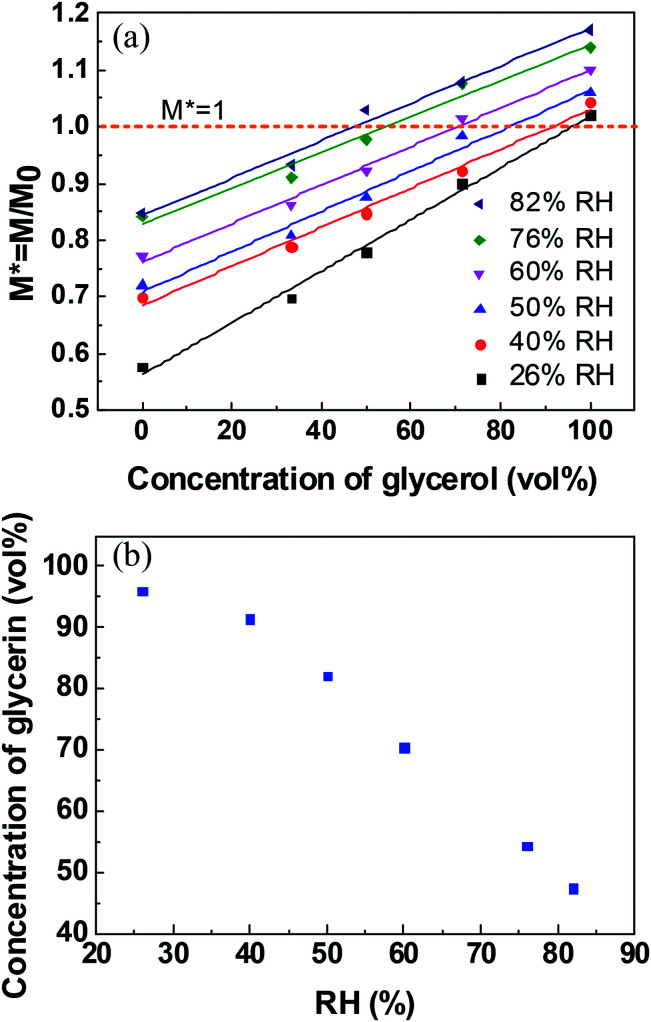
(a) Correlation between normalized mass and glycerol concentrations of liquid marbles at 20 min at different RHs. (b) The correlation of relative humidity and glycerol volume concentrations for a long-term liquid marble.

The correlation between normalized mass and volume concentrations of glycerol within the liquid marble at 20 min is plotted in Fig. 4a. It clearly shows a linear relationship between normalized mass and glycerol volume concentrations at the same RH. M* = 1 indicates that the mass of the liquid marble did not change with time and a liquid marble with long-term stability is formed. Thus, the volume concentration of glycerol in stable water/glycerol marbles can be extracted from the intersection when M* = 1 in Fig. 4a. The volume concentration of glycerol with respect to RH for a stable liquid marble is shown in Fig. 4b. To maintain long-term stability, the volume concentrations of glycerol in the liquid marble should be 96.0% and 47.4% when the RH values are 26% and 82%, respectively. As RH increased, the volume concentration of glycerol in the liquid marble decreased.

**Fig. 5:**
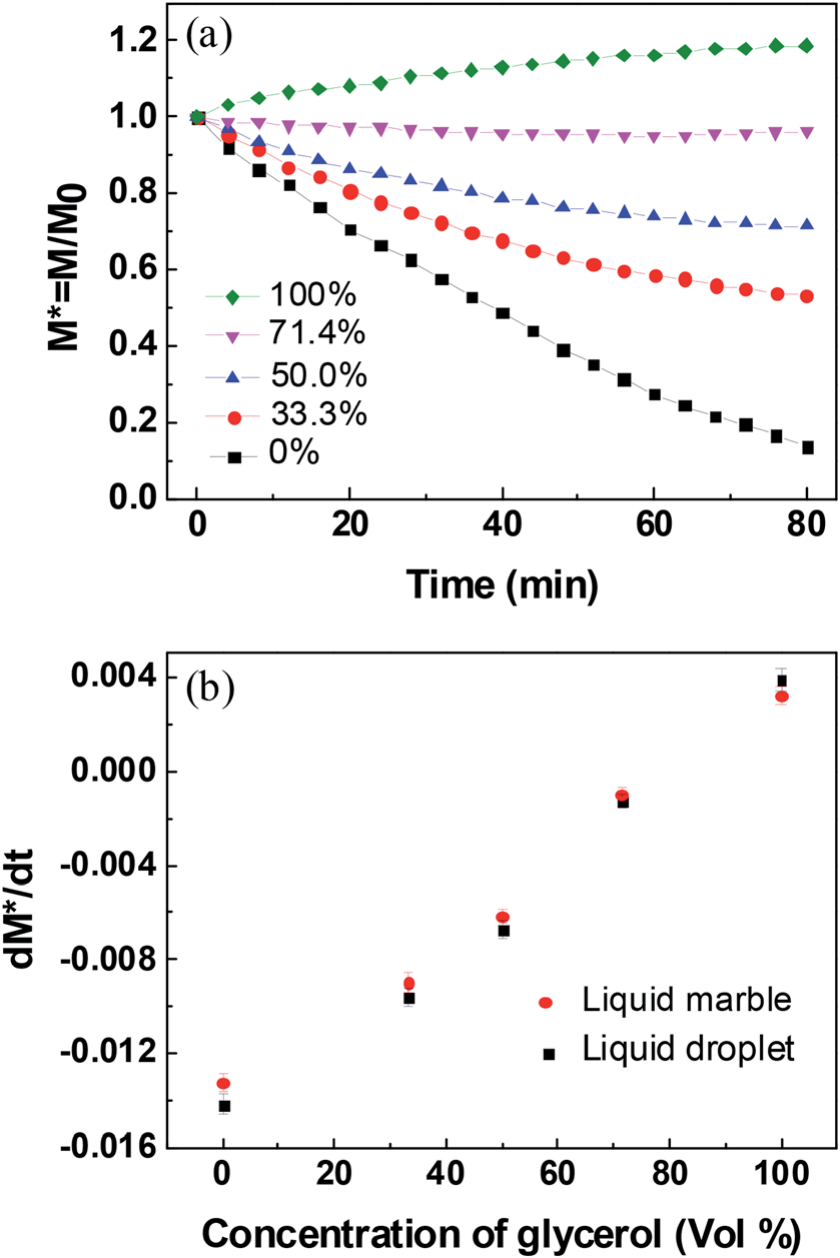
(a) Normalized mass of naked aqueous glycerol droplets with different glycerol concentrations at 50% RH. (b) Evaporation/absorption rate of a liquid marble and a liquid droplet with different glycerol concentrations at 50% RH.

To compare the evaporation/absorption rates (dM*/d*t*) of the liquid droplet covered with or without PFC nanoparticles, the mass changes of naked droplets with different glycerol concentrations at 50% RH were examined. It should be pointed out that the water uptake of the PFC nanoparticles can be considered ignorable when compared to water evaporation/adsorption. Under the RH of 26%, 40%, 50%, 60%, 76%, and 82%, the water uptake of the PFC nanoparticles was about 0.00%, 0.02%, 0.03%, 0.05%, 0.08%, and 0.09%, respectively (Fig. S2 in ESI†). Therefore, for a water marble composed of 1 mg of PFC nanoparticles and 10 mg of liquid, the extremely low water uptake of the PFC nanoparticles had negligible influence on the mass change of the liquid marble. Fig. 5a shows the plots of the normalized mass of naked droplets with time. As expected, liquid droplets with higher glycerol concentrations have lower evaporation rates, which are similar to that of liquid marbles. Fig. 5b compares the water evaporation/absorption rates for the liquid droplets covered with or without PFC nanoparticles. The liquid marbles with glycerol concentrations lower than 71.4% showed lower evaporation rates than the corresponding liquid droplets. The difference in the evaporation rates of the liquid droplets and liquid marbles enhanced as the glycerol content decreased. However, the absorption rate of the pure glycerol marbles was lower than that of the pure glycerol droplets. Obviously, the difference in the water evaporation/absorption rates of the naked liquid droplet and liquid marble was caused by the multilayered PFC nanoparticles, which prevented the water molecules from passing through the liquid/air interface.

The evaporation resistance of aqueous glycerol marbles, *φ*, is further discussed. *φ* can be given by^35,44^2
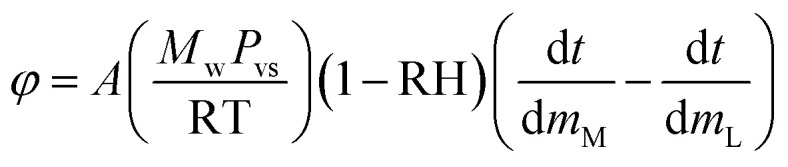
3
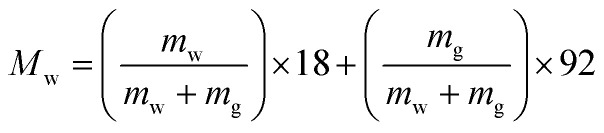
4
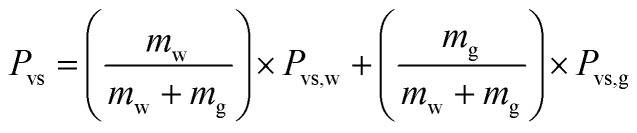
where *A* is the initial surface area of the evaporating material with an assumed spherical cap shape, *R* is the gas constant, *M*_w_ is the average molecular weight of the aqueous glycerol droplet, *m*_w_ and *m*_g_ are the masses of water and glycerol in aqueous glycerol droplets, respectively; *P*_vs_ is the saturation vapor pressure of aqueous glycerol, *P*_vs,g_ and *P*_vs,w_ are the saturation vapor pressures of glycerol (0.02 Pa) and water (3.178 kPa) at 25 °C, respectively, *m*_M_ is the mass of the liquid marble and *m*_L_ is the mass of the naked liquid droplet.

**Fig. 6:**
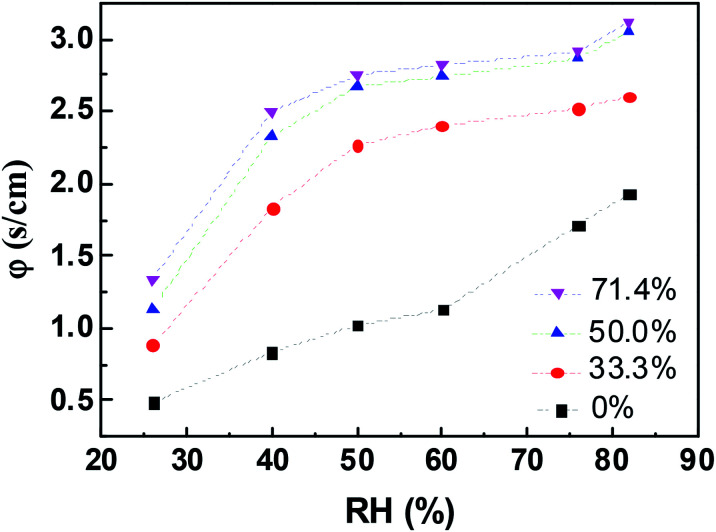
Variation in the evaporation resistance, *φ*, of aqueous glycerol marbles with various RHs.

The change in *φ* in aqueous glycerol marbles as a function of RH was calculated using eqn (2), and the result is shown in Fig. 6. The liquid marbles exhibited higher resistance as the glycerol concentration increased. The self-assembled PFC particles at the liquid–air interface of the liquid marble produced a certain effect on reducing the evaporation rate compared with that for the liquid droplet. For water marbles, with the increase in RH from 26% to 82%, the *φ* values increased from 0.488 to 1.931. When RH was low, the water molecules easily escaped from the liquid marble due to the high driving energy, and the PFC nanoparticles had a lower screening effect^36^ for water to enter in/out of the droplet.

## Conclusions

In summary, stable liquid marbles were fabricated by encapsulating glycerol/water droplets using superhydrophobic cellulose nanocrystals. The effect of water evaporation/absorption on the stability of aqueous glycerol marbles at different relative humidities was studied. For the liquid marble with the same glycerol volume concentration, the evaporation rate of water decreased with the increase in RH. The evaporation/absorption rates of liquid marbles decreased with the increase in glycerol concentrations at the same RH. Compared to naked liquid droplets, liquid marbles exhibited higher evaporation/absorption resistance due to the multilayered coating. When the evaporation rate and absorption rate of the coated mixture solution reached a balance, the stable liquid marbles could be potentially used as miniature reactors for long-standing aqueous chemical reactions.

## Conflicts of interest

There are no conflicts to declare.

## Supplementary Material

RA-009-C9RA05728E-s001

## References

[cit1] Aussillous P., Quere D. (2001). Nature.

[cit2] Aussillous P., Quere D. (2006). Proc. R. Soc. A.

[cit3] McHale G., Newton M. I. (2011). Soft Matter.

[cit4] Fujii S., Yusa S.-i., Nakamura Y. (2016). Adv. Funct. Mater..

[cit5] Oliveira N. M., Reis R. L., Mano J. F. (2017). Adv. Healthcare Mater..

[cit6] Matsukuma D., Watanabe H., Wu H., Ogawa S., Jinnai H., Takahara A. (2017). Kobunshi Ronbunshu.

[cit7] Matsukuma D., Watanabe H., Yamaguchi H., Takahara A. (2011). Langmuir.

[cit8] Ogawa S., Watanabe H., Wang L., Jinnai H., McCarthy T. J., Takahara A. (2014). Langmuir.

[cit9] Cengiz U., Erbil H. Y. (2013). Soft Matter.

[cit10] Wu H., Watanabe H., Ma W., Fujimoto A., Higuchi T., Uesugi K., Takeuchi A., Suzuki Y., Jinnai H., Takahara A. (2013). Langmuir.

[cit11] Ooi C. H., Vadivelu R. K., St John J., Dzung Viet D., Nam-Trung N. (2015). Soft Matter.

[cit12] Zhou X., Lin X., White K. L., Lin S., Wu H., Cao S., Huang L., Chen L. (2016). Cellulose.

[cit13] Mei K. K., Ooi C. H., Mohd-Yasin F., Nguyen A. V., Evans G. M., Nguyen N. T. (2017). Microfluid. Nanofluid..

[cit14] Bormashenko E., Frenkel M., Bormashenko Y., Chaniel G., Valtsifer V., Binks B. P. (2017). Langmuir.

[cit15] Zhao Y., Fang J., Wang H. X., Wang X. G., Lin T. (2010). Adv. Mater..

[cit16] Hu Y., Jiang H., Liu J., Li Y., Hou X., Li C. (2014). RSC Adv..

[cit17] Kavokine N., Anyfantakis M., Morel M., Rudiuk S., Bickel T., Baigl D. (2016). Angew. Chem., Int. Ed..

[cit18] Lin X., Ma W., Wu H., Cao S., Huang L., Chen L., Takahara A. (2016). Chem. Commun..

[cit19] Lin X., Ma W., Chen L., Huang L., Wu H., Takahara A. (2018). Soft Matter.

[cit20] Geyer F., Asaumi Y., Vollmer D., Butt H.-J., Nakamura Y., Fujii S. (2019). Adv. Funct. Mater..

[cit21] Kawashima H., Shioi A., Archer R. J., Ebbens S. J., Nakamura Y., Fujii S. (2019). RSC Adv..

[cit22] Ma W., Wu H., Higaki Y., Takahara A. (2018). Chem. Rec..

[cit23] Planchette C., Biance A. L., Pitois O., Lorenceau E. (2013). Phys. Fluids.

[cit24] Matsukuma D., Watanabe H., Fujimoto A., Uesugi K., Takeuchi A., Suzuki Y., Jinnai H., Takahara A. (2015). Bull. Chem. Soc. Jpn..

[cit25] Zhao Y., Xu Z., Niu H., Wang X., Lin T. (2015). Adv. Funct. Mater..

[cit26] Jin J., Ooi C. H., Dzung Viet D., Nam-Trung N. (2018). Soft Matter.

[cit27] Liu Z., Fu X. Y., Binks B. P., Shum H. C. (2017). Soft Matter.

[cit28] Chu Y., Wang Z., Pan Q. (2014). ACS Appl. Mater. Interfaces.

[cit29] Sheng Y., Sun G., Wu J., Ma G., Ngai T. (2015). Angew. Chem., Int. Ed..

[cit30] Luo X., Yin H., Li X. e., Su X., Feng Y. (2018). Chem. Commun..

[cit31] Rong X., Ettelaie R., Lishchuk S. V., Cheng H., Zhao N., Xiao F., Cheng F., Yang H. (2019). Nat. Commun..

[cit32] Liu Z., Yang T., Huang Y., Liu Y., Chen L., Deng L., Shum H. C., Kong T. (2019). Adv. Funct. Mater..

[cit33] Laborie B., Lachaussée F., Lorenceau E., Rouyer F. (2013). Soft Matter.

[cit34] Bhosale P. S., Panchagnula M. V., Stretz H. A. (2008). Appl. Phys. Lett..

[cit35] Tosun A., Erbil H. Y. (2009). Appl. Surf. Sci..

[cit36] Dandan M., Erbil H. Y. (2009). Langmuir.

[cit37] Doganci M. D., Sesli B. U., Erbil H. Y., Binks B. P., Salama I. E. (2011). Colloids Surf., A.

[cit38] Matsukuma D., Watanabe H., Minn M., Fujimoto A., Shinohara T., Jinnai H., Takahara A. (2013). RSC Adv..

[cit39] Ooi C. H., Bormashenko E., Nguyen A. V., Evans G. M., Dao D. V., Nguyen N.-T. (2016). Langmuir.

[cit40] Fullarton C., Draper T. C., Phillips N., Mayne R., de Lacy Costello B. P. J., Adamatzky A. (2018). Langmuir.

[cit41] Sreejith K. R., Ooi C. H., Dao D. V., Nguyen N.-T. (2018). RSC Adv..

[cit42] Nguyen T. H., Hapgood K., Shen W. (2010). Chem. Eng. J..

[cit43] Kendall K. (1994). Science.

[cit44] La Mer V. K., Healy T. W. (1965). Science.

