# Supplementary material for: Influence of water evaporation/absorption on the stability of glycerol–water marbles
Source: RSC Adv. 2019 Oct 28;9(59):34465–71. doi: 10.1039/c9ra05728e (PMC9073916; doi:10.1039/c9ra05728e)
Supplement: RA-009-C9RA05728E-s001 [file RA-009-C9RA05728E-s001.pdf]

Electronic Supplementary Material (ESI) for RSC Advances.
This journal is © The Royal Society of Chemistry 2019

Supplementary Material (ESI) for **

Supplementary Information

Influence of water evaporation/absorption on the stability of glycerol-water marbles

Xinxing Lin,[a] Wei Ma,[b,c] Lihui Chen,[a,*] Liulian Huang,[a] Hui Wu,[a,*]and Atsushi Takahara[b,c]

[a]*College of Material Engineering, Fujian Agriculture and Forestry University, Fuzhou, 350002, China*

[b]*Institute for Materials Chemistry and Engineering, Kyushu University, 744 Motooka, Nishi-ku, Fukuoka 819-0395, Japan*

[c]*International Institute for Carbon-Neutral Energy Research (WPI-I2CNER), Kyushu University 744, Motooka, Nishi-ku, Fukuoka 819-0395, Japan*

Experimental setup for the investigation of the morphological evolution of liquid marble

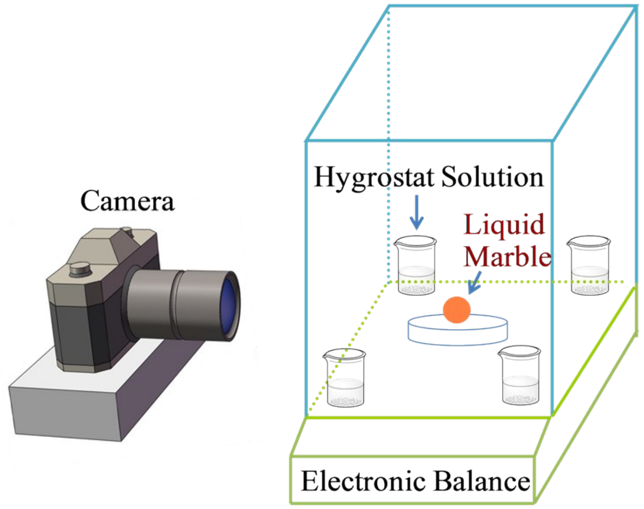

Fig. S1 Schematic of the experimental setup for the investigation of the morphological evolution of liquid marble.

The gravimetric measurements of liquid marbles with different glycerol concentrations under various RH were conducted using an electric balance (AUW120D). The electric balance was placed in an air-conditioned laboratory at 297.2 ± 1.0 K under RH of 50 ± 2 %. The geometry of liquid marbles was

acquired by a camera (Nikon V3). The liquid marble was placed on the weighting pan of a sealed electronic balance. Four bottles of same saturated solution were placed within the corner of electronic balance to adjust humidity.

Water uptake of poly(DOPAm-co-PFOEA)/Fe_3O_4/CNC (PFC) nanoparticles

To evaluate the water uptake of PFC nanoparticles, about 0.1 g of sample was placed in a closed electronic balance under different RH. The mass of the PFC nanoparticles was recorded after 2 h. The water uptake of PFC nanoparticles with various RH was shown in Fig. S2.

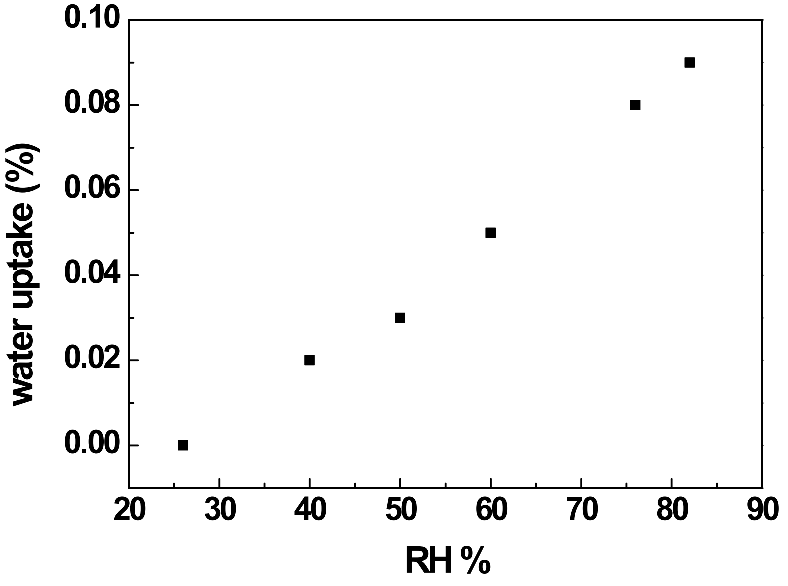

Fig. S2 The water uptake of PFC nanoparticles with different RH.
